# *Coxiella burnetii* establishes a small cell variant (SCV)-like persistent form to survive adverse intracellular conditions

**DOI:** 10.1080/21505594.2026.2707792

**Published:** 2026-09-10

**Authors:** Faiza Asghar, Inaya Hayek, Elke Bachmann, Christian Berens, Elisabeth Liebler-Tenorio, Anja Lührmann

**Affiliations:** a Mikrobiologisches Institut, Klinische Mikrobiologie, Immunologie und Hygiene, Universitätsklinikum Erlangen, Friedrich-Alexander-Universität (FAU) Erlangen-Nürnberg, Erlangen, Germany; b Friedrich-Loeffler Institut, Institut für molekulare Pathogenese, Jena, Germany; c FAU Profile Center Immunomedicine (FAU I-MED), Friedrich-Alexander University Erlangen-Nürnberg, Erlangen, Germany

**Keywords:** *Coxiella burnetii*, persistence, hypoxia, SCV, antibiotic persistence, ScvA

## Abstract

*Coxiella burnetii* is an obligate intracellular zoonotic bacterium that causes Q fever. Infections can be either acute or chronic. Of note, chronic Q fever can develop months or years after primary infection without clinical symptoms, suggesting bacterial persistence. Yet, information about the induction, regulation, and/or location of *C. burnetii* persistence is rare. We have shown that during infection of primary macrophages, hypoxia-induced citrate limitation results in inhibition of *C. burnetii* replication without affecting viability. Here, primary murine macrophages were infected with *C. burnetii* under normoxic (21% O_2_) and hypoxic (0.5% O_2_) conditions to clarify how *C. burnetii* survives this environmental stress condition. Our data suggest that under hypoxic conditions, *C. burnetii* does not undergo the stringent response, but instead enters an SCV-like form, which is smaller in size, possesses condensed chromatin material and a thicker cell wall. These changes have functional consequences, as the SCV-like persistent form of *C. burnetii* is more infectious, more tolerant to antibiotics and less sensitive to clearance by IFNγ activated macrophages. Hence, the development of the SCV-like persistent form of *C. burnetii* prevents elimination of the pathogen, which in turn allows the pathogen to thrive once the conditions again change in its favor.

## Introduction

*Coxiella burnetii* is a Gram-negative, non-motile, obligate intracellular, pleomorphic coccobacillus [1]. The bacterium is highly resistant to different stresses, including osmotic shock, high temperature, desiccation, or UV light, providing environmental stability. This stability is attributed to the small-cell variant (SCV) form, which develops during the biphasic development cycle [2]. It is characterized by a rod-shaped size of 0.2 to 0.5 µm in length (in negative contrast preparations), condensed chromatin, and a dense cell wall [3]. Moreover, SCVs display little metabolic activity [2]. Within a host cell, the SCV differentiates into the large-cell variant (LCV), which can exceed 1 µm in length (in negative contrast preparations) and represents the replication-competent form of *C. burnetii* [3]. The transition from LCV to SCV occurs during the shift from the exponential to the stationary phase of intracellular growth [4].

*C. burnetii* is found worldwide, with the exception of New Zealand. It infects a vast diversity of species, ranging from ticks, fish, birds, and rodents to mammals, including ruminants and humans [5]. In humans, it causes the anthropozoonotic disease Q fever [1]. There are two forms of Q fever, acute and chronic Q fever. Acute Q fever mainly presents as a flu-like illness, atypical pneumonia, or hepatitis. It is either self-limiting or resolves after treatment with doxycycline for two weeks. Chronic Q fever develops months or even years after the initial infection in 2–5% of cases, suggesting a phase of bacterial persistence before the development of disease. Its most common manifestation is endocarditis. There are several risk factors for the development of chronic Q fever, such as male gender, preexisting valvular conditions, or contact with farm animals [6,7]. Without treatment, chronic Q fever might be fatal. Therefore, early treatment with doxycycline and hydroxychloroquine for at least 18 months is required [8,9].

To survive adverse conditions, bacteria can enter a state of persistence. Bacterial persistence is a transient, non-inheritable, and quiescent state. Importantly, the persistent bacterium returns to normal growth when the condition changes. Thereby, bacterial persistence is associated with relapsing infections. Bacteria encode different regulatory programs to coordinate persistence, including the general stress response or the stringent response [10–12]. The stringent response is a stress adaptation program, which is mediated by the alarmones (p)ppGpp to reprogram transcription, translation, and metabolism [13]. The alarmone levels are controlled by the RelA/SpoT homologue superfamily (RSH), which either synthesizes or degrades (p)ppGpp [14]. Thus, under amino acid starvation, RelA forms a complex with deacylated tRNA, which activates the (p)ppGpp synthetic activity of RelA [15]. SpoT is only a weak (p)ppGpp synthetase, but its strong hydrolytic activity is essential for balancing (p)ppGpp levels [16]. Importantly, activation of RelA and SpoT is controlled by heterologous protein interactions and, potentially, oligomerization [16,17]. In addition, the binding of Mn^2+^ and Mg^2+^ to the active center of SpoT is believed to activate its hydrolytic activity [18]. The alarmones (p)ppGpp, together with the transcription factor DksA, induce synthesis of the alternative sigma factor RpoS, which allows the pathogen to adapt to the stress situation [19]. RpoS levels in the cell are controlled at the transcriptional, translational, and proteolytic levels [20].

*C. burnetii* DNA was detected years after acute Q fever in 65% of the bone marrow aspirates analyzed, indicating the presence of residual bacteria, which might represent the seed for recrudescent infection and the development of chronic Q fever [21]. In addition to bone marrow, adipose tissue was suggested as a site of *C. burnetii* persistence [22,23]. Neither the location nor the molecular mechanism(s) resulting in *C. burnetii* persistence are completely understood [11]. Diverse stimuli hinder bacterial replication, such as IFNγ, TNFα, or iron limitation [24,25]. However, whether these stimuli induce *C. burnetii* persistence is unknown. *C. burnetii* possesses genes involved in the general stress response and in the stringent response [26], which might be involved in the development of persistent *C. burnetii*.

Here, we investigated whether hypoxia-mediated alterations of host cell metabolism induce *C. burnetii* persistence. Indeed, our data indicate that under hypoxia, and thus under citrate-limiting conditions, *C. burnetii* establishes an SCV-like persistent form, which is characterized by condensed chromatin, an altered cell wall, increased infectivity, and antibiotic tolerance. In addition, we provide evidence that the development of *C. burnetii* persistence might not be mediated by the stringent response, but rather by a general stress response.

## Results

**Figure 1.**
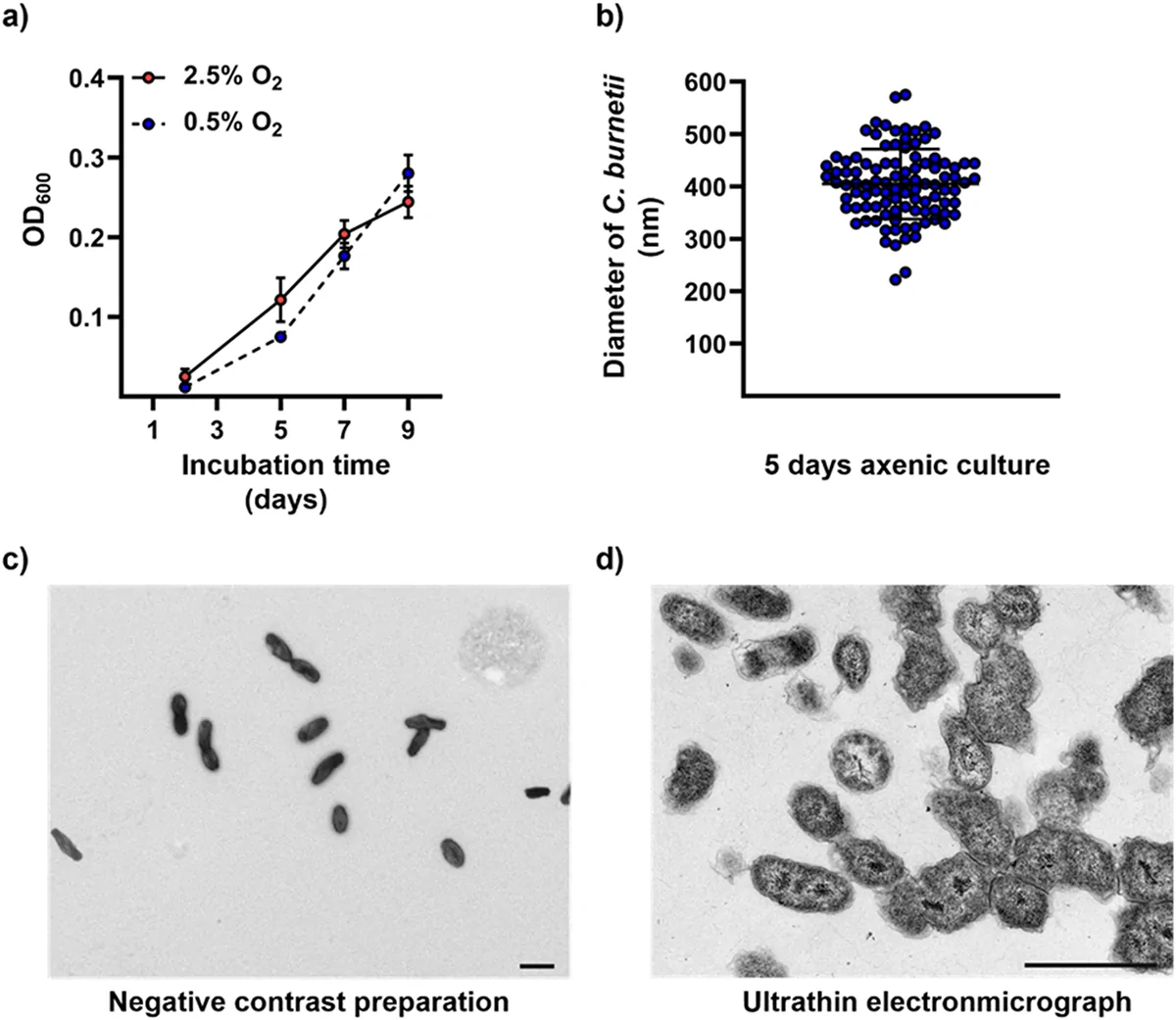
The 5-day axenic *C. burnetii* culture mainly contains large cell variants (LCVs). *C. burnetii* was grown under axenic conditions at 2.5% or 0.5% O_2_ for 9 days. a) The OD_600_ values were determined on the day indicated. Each dot represents the mean of three independent experiments performed in duplicates. Data are shown as mean ± SD. b) the bacterial size was determined from electron microscopy (EM) images of a 5-day axenic culture of *C. burnetii* grown at 2.5% O_2_. The diameters of 100 bacteria from two independent preparations were measured after negative contrast preparation. Each dot represents an individual bacterium. Data are shown as mean ± SD. c) The negative contrast preparation of the 5-day axenic culture of *C. burnetii* grown at 2.5% O_2_ shows rather uniform, plump rods. d) Ultrathin sections of the same samples revealed characteristic LCV morphology, including electron-lucent periplasmic spaces and mostly homogeneous distribution of chromatin. Scale bars are 1 µm.

**Severe hypoxia does not influence axenic replication of** ***C.*** ***burnetii.** C.* *burnetii* is cultivated axenically at 37°C, 5% CO_2_ and 2.5% O_2_ [27]. In order to verify that 0.5% O_2_, the hypoxic condition used in this study, does not prevent axenic replication of *C. burnetii*, we cultivated the bacteria in ACCM-2 media under 0.5% and 2.5% O_2_ for 9 days. As shown in Figure 1(a), *C. burnetii* replicated equally well under both conditions, indicating that severe hypoxia at 0.5% O_2_ did not affect *C. burnetii* viability or replication. In addition, no difference in infectivity was observed (Fig. S1). We used *C. burnetii* grown for 5 days in ACCM-2 media before infecting the respective host cells. It has been shown by cryo-electron tomography imaging that day 5 axenic cultures mainly represent LCVs [28]. Similarly, we mainly found LCVs in our 5-day axenic culture (Figures. 1(b-d)). Consequently, LCV of *C. burnetii* were used in the following infection experiments.

**Hypoxia triggers a** **general stress response, but not the stringent response in intracellular** ***C.*** ***burnetii***. We have previously shown that *C. burnetii* stays viable, but is unable to replicate in hypoxic macrophages [29,30]. To investigate whether hypoxia-mediated citrate-limitation results in *C. burnetii* persistence, we characterized *C. burnetii* at 48 hours post-infection in normoxic and hypoxic bone marrow-derived macrophages (BMDM) at the transcriptional level. This time point was chosen, as *C. burnetii* has not yet started to replicate in BMDM in our hands (Fig. S2) [29]. It should be noted that other reports demonstrate *C. burnetii* replication in BMDM at this time point [31,32]. Importantly, we did not observe a compromised viability of the infected BMDM within the time period analyzed (data not shown).

**Figure 2.**
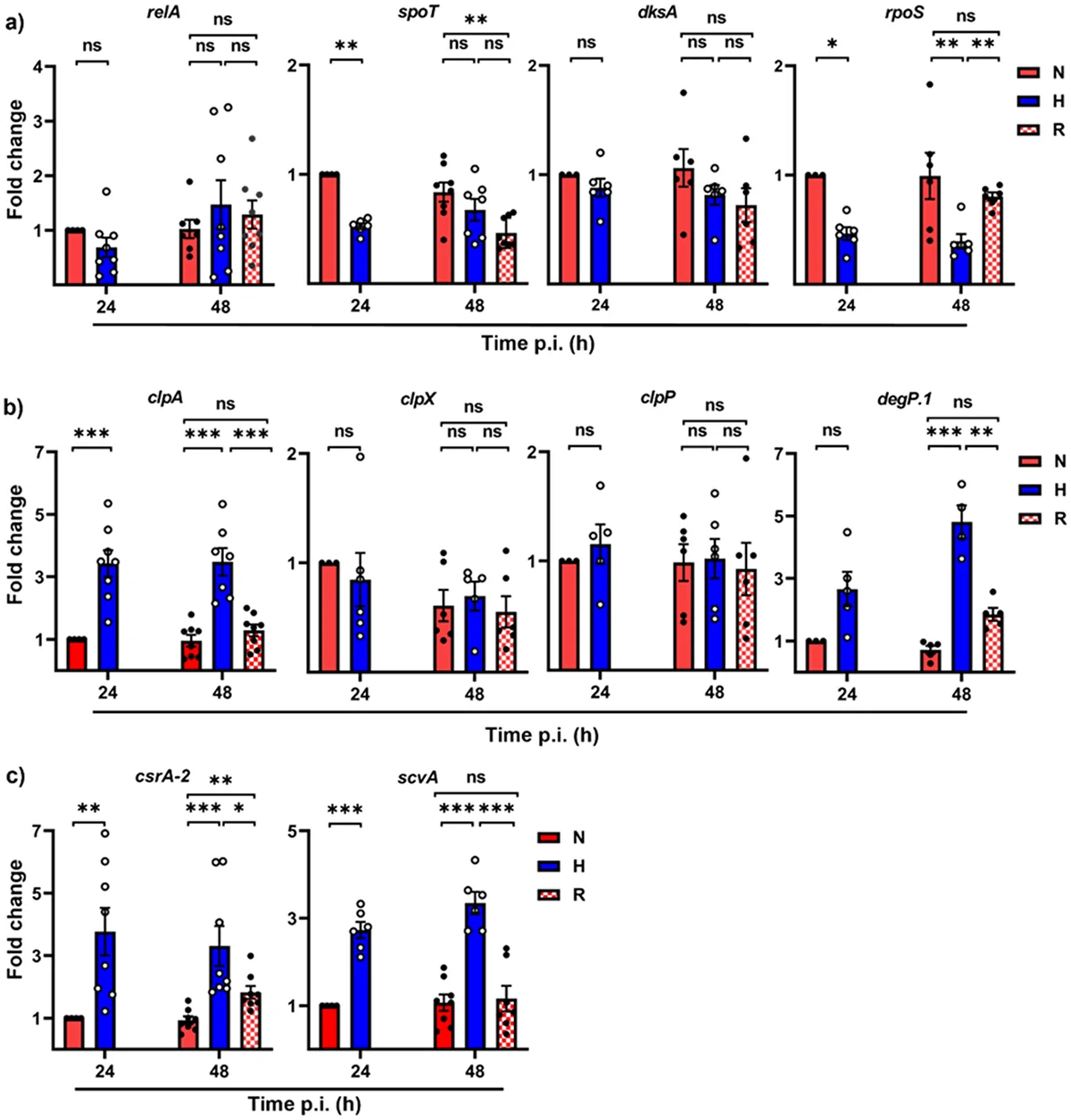
Hypoxia-mediated changes in infected BMDM trigger a general stress response in *C. burnetii*. Murine BMDM were infected for 48 h with *C. burnetii* at a multiplicity of infection of 10 under normoxia (N), hypoxia (H) or re-oxygenated (R) after 24 h of hypoxia. Using qRT-PCR, the gene expression of bacterial genes involved in a) the stringent response, b) a general stress response and c) carbon storage regulator (csrA-2) and *scvA* expression was determined. The data are depicted as mean ± SEM of 2^−ΔΔCT^ fold change values (using IS1111 as a calibrator) and normalized to a 24 h normoxic sample. Each dot represents the data from 3 – 4 independent experiments performed in duplicates. One-sample t-test and Mann‑Whitney U test, ***p < 0.001, **p < 0.01, *p < 0.05, ns = not significant.

Bacterial persistence is reversible. Consequently, a reoxygenation control (24 hours hypoxia followed by 24 hours normoxia) was included. First, we analyzed the expression level of genes involved in the stringent response, which is a ubiquitous stress response to nutrient starvation [33]. A hallmark of the stringent response is the accumulation of the alarmones guanosine tetraphosphate (ppGpp) and pentaphosphate (pppGpp). Their levels are controlled by RelA (CBU_1375) and SpoT (CBU_0303) [34]. The alarmones together with the transcription factor DksA (CBU_1696) interact with RNA polymerase to promote binding to the sigma factor RpoS (CBU_1669), thereby shifting transcription and adaptation to the stress situation [19]. As shown in Figure 2(a), neither *relA* nor *dksA* is transcriptionally modulated by oxygen limitation. For *spoT* and *rpoS*, reduced mRNA levels were observed under hypoxic conditions. These data suggest that hypoxia might not induce the stringent response in *C. burnetii*.

As *C. burnetii* lacks a type II toxin-antitoxin system, which is linked to the development of persistence in other bacterial pathogens [11], we concentrated on the expression of genes involved in the general stress response. Clp family proteases have multiple functions, including regulation of stress responses [35]. *C. burnetii* encodes the ATPase subunits ClpA (CBU_1196) and ClpX (CBU_0739) as well as the proteolytic subunit ClpP (CBU_0738). ClpA and ClpX are chaperones that bind ClpP to establish ClpAP or ClpXP, which allows the efficient processing of large peptides and folded proteins [35]. Similarly, the periplasmic dual-function protease DegP.1 (CBU_0176) is critical for the bacterial stress response by degrading a wide range of proteins [36]. The expression levels of *clpA* and *degP.1* were increased in hypoxic BMDM. Importantly, this upregulation was revoked after reoxygenation, demonstrating the reversible nature of this bacterial response (Figure 2(b)). The carbon storage regulator (CsrA, CBU_1050) binds mRNA and thereby globally regulates gene expression in a post-transcriptional manner [37]. The expression of *csrA-2* was upregulated 3- to 4-fold under hypoxic conditions (Figure 2(c)). Although the function of CsrA-2 has not been investigated in detail in *C. burnetii*, it has been suggested that it might perform a global regulatory function in the SCV form [38]. Hence, the expression level of *scvA* was determined. ScvA (CBU_1267.1) is a small peptide, which is mainly expressed in the SCV form [39]. Indeed, *scvA* is upregulated in infected hypoxic BMDM. After reoxygenation, this upregulation was abrogated (Figure 2(c)). Together, these data indicate that the hypoxic environment within murine BMDM triggers a general stress response rather than a stringent response in *C. burnetii*. Furthermore, the stress induced by the intracellular environment in hypoxic BMDM might trigger the transition from LCV to SCV.

**Figure 3.**
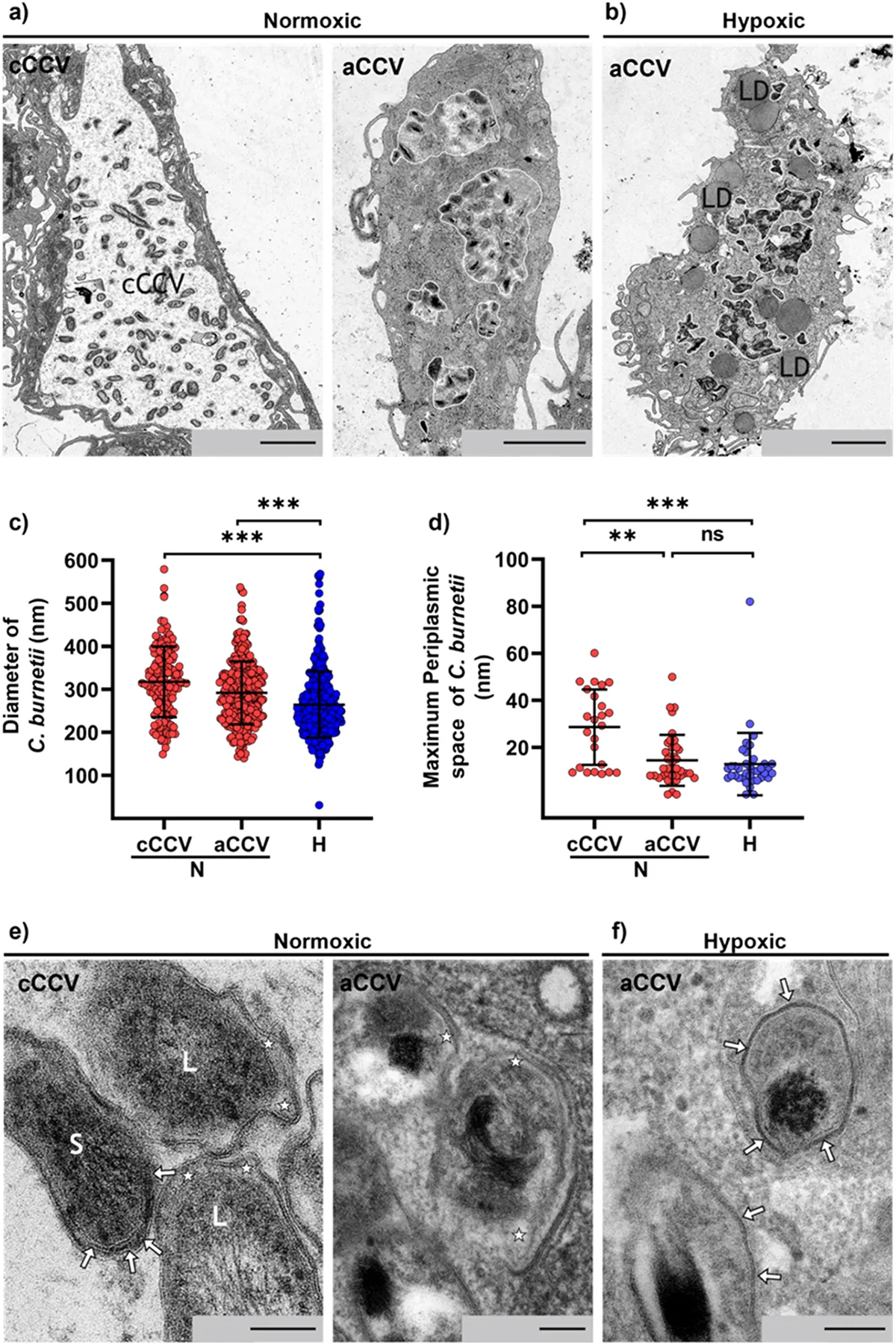
Hypoxia-mediated changes in infected BMDM induce morphological changes in *C. burnetii*. Murine BMDM were infected for 48 h with *C. burnetii* at MOI 10 under normoxia (N) or hypoxia (H). The cells were fixed with ruthenium red glutaraldehyde, embedded in araldite, and ultrathin sectioned for electron microscopy. a) *C. burnetii* infected normoxic (N) BMDM with a characteristic CCV (cCCV) and another cell with multiple abnormal CCVs (aCCVs), indicated by white outlines, and b) a hypoxic (H) BMDM with multiple aCCVs (indicated by white outlines) are shown. Lipid droplets are marked with LD. The scale bar = 2 µm. c) The diameter of 140 bacteria in cCCV and 300 bacteria in aCCV of normoxic and 300 bacteria in hypoxic BMDM were measured. The data is depicted as mean ± SD from 2–3 independent experiments. Mann‑Whitney U test, ***p < 0.001. d) The maximal thickness of the periplasmic space was measured from at least 30 bacteria of each condition. Shown are the means ± SD, Mann‑Whitney U test, ***p < 0.001. e) Morphology of the bacterial wall of *C. burnetii* in BMDM cells is shown: two LCVs (L) with irregularly dilated electron lucent periplasmic spaces (*) and one SCV (S) with narrow, electron dense periplasmic space (arrows) in a cCCV under normoxic conditions (N-cCCV), three pleomorphic *C. burnetii* with LCV-like periplasmic spaces (*) in an aCCV under normoxic conditions (N-aCCV), and f) two bacteria with SCV-like periplasmic spaces (arrows) under hypoxic conditions. The scale bar = 100 nm.

**Hypoxia leads to morphological changes in intracellular** ***C.*** ***burnetii***. To determine the developmental stage of *C. burnetii*, the morphology of bacteria in normoxic and hypoxic BMDM was analyzed using TEM (Figures. 3(a,b)). Under hypoxic conditions, BMDM had an increased number of peroxisomes and/or lipid bodies (Figures. 3(b) and S3), which might indicate a hypoxic stress situation [40]. Importantly, the occurrence of peroxisomes and lipid bodies in uninfected hypoxic cells has been described previously [41]. In hypoxic BMDMs, mainly lipid bodies were present, whereas peroxisomes were observed frequently. Both characteristic CCVs (cCCV), which contained predominantly LCVs and only a few SCVs, and abnormal CCVs (aCCV) were seen in normoxic BMDM (Figure 3(a)). While aCCVs were already very numerous under normoxic conditions (Fig. S4), they were present almost exclusively under hypoxic conditions, often forming multiple smaller CCVs (Figure 3(b)). *C. burnetii* in aCCV were often crowded closely together and highly pleomorphic, which did not allow a clear discrimination of LCV and SCV. Thin, electron-dense bacteria were more frequent, but bacteria with large central rod-shaped electron-dense condensation and swollen bacteria with partly clumped, partly lytic cytoplasm were also seen. The mean diameter of *C. burnetii* in hypoxic BMDM was lower compared to normoxic BMDM indicating a higher frequency of SCV-like forms (Figure 3(c)). In addition to the size difference, LCVs and SCVs can be discriminated by the morphology of the bacterial cell wall, especially the periplasmic space (Figure 3(d)). As observed in cCCVs under normoxic conditions, the LCV periplasmic space is electron-lucent and irregularly dilated resulting in an undulating outer membrane, whereas in SCV, it is rather uniform, narrow, and filled with an electron dense matrix (Figure 3(e)), similar as described previously [28,42]. 36% of the pleomorphic *C. burnetii* in aCCVs under normoxic conditions and 73% of bacteria under hypoxic conditions had SCV-like periplasmic spaces. These data indicate that even normoxic murine BMDM might represent a stressful environment for *C. burnetii*. Importantly, hypoxic conditions boost the development of aCCVs and of the SCV-like form of *C. burnetii* (Figure 3(f)).

**Figure 4.**
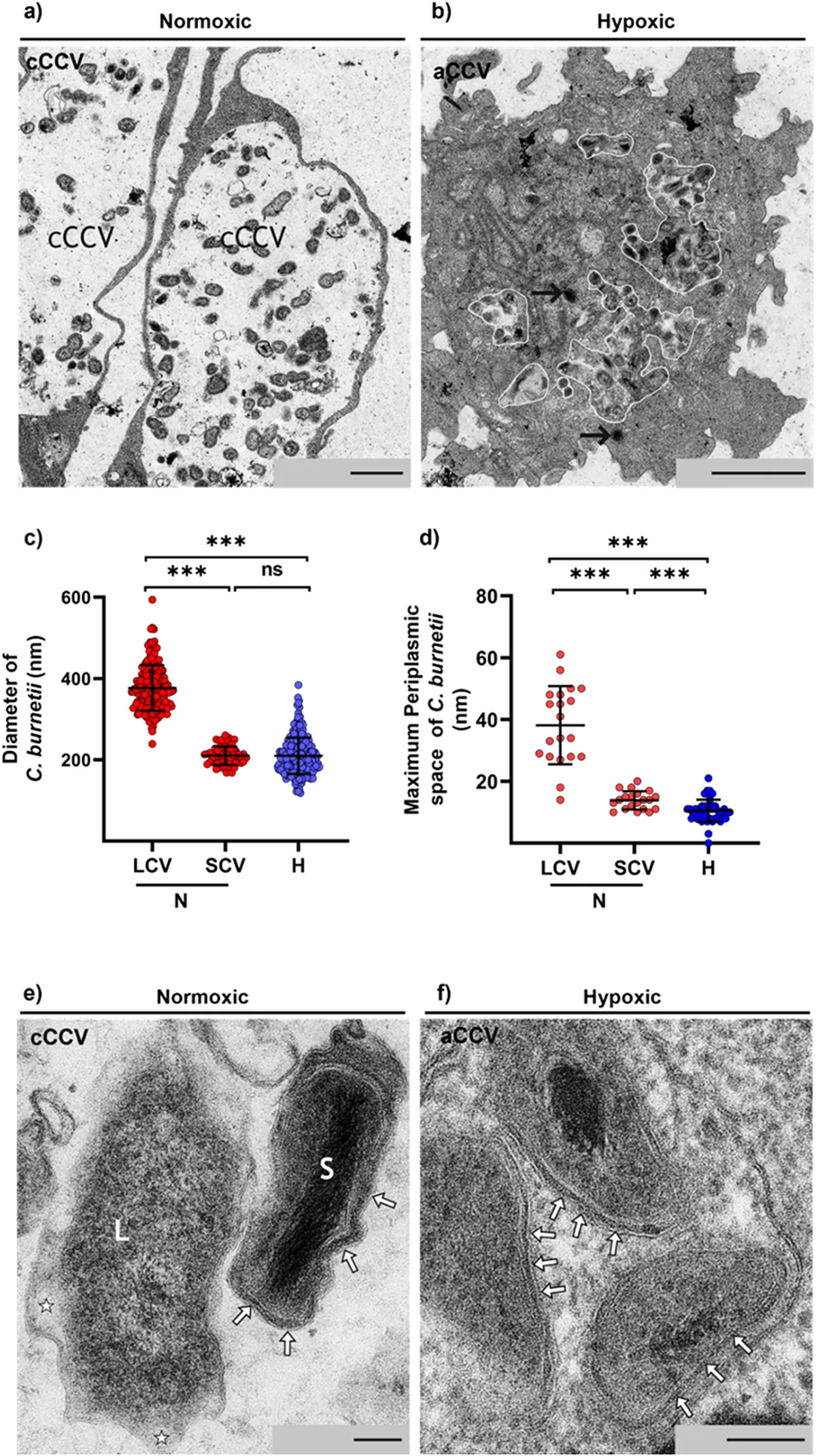
Hypoxia-mediated changes in infected human endothelial cells induce morphological changes in *C. burnetii*. Human EA.hy926 cells were infected for 48 h with *C. burnetii* at MOI 200 under normoxia (N) or hypoxia (H). The cells were fixed with ruthenium red glutaraldehyde, embedded in araldite and ultrathin sectioned for electron microscopy (EM). a) Two *C. burnetii* infected normoxic (N) cells with large cCCV and b) a hypoxic (H) EA.hy926 cell with multiple small aCCV (indicated by white outlines) are shown. Peroxisomes were highlighted by arrows. The scale bar = 2 µm. c) The size of 250 bacteria per condition from 2–3 independent experiments were measured. The data are depicted as mean ± SD. Mann‑Whitney U test, ***p < 0.001. d) The thickness of the periplasmic space was measured from 40 bacteria (20 LCV and 20 SCV) under normoxic and 40 bacteria under hypoxic conditions. Shown are the means ± SD, t-test. ***p < 0.001. e) Morphology of the bacterial cell wall of *C. burnetii* in an EA.hy926 cell is shown: one LCV (L) with irregularly dilated electron lucent periplasmic space (*) and one SCV (S) with narrow, electron dense periplasmic space (arrows) under normoxic (N) conditions and f) three pleomorphic bacteria with SCV-like periplasmic spaces (arrows) under hypoxic conditions. The scale bar = 100 nm.

To verify that the development of SCV-like *C. burnetii* is not only restricted to hypoxic murine BMDM, *C. burnetii* grown in the human endothelial cell line EA.hy926 was analyzed (Figure 4(a,b)). As in BMDM, increased numbers of peroxisomes and occasionally lipid bodies indicated hypoxic stress (Figure 4(b)). Normoxic EA.hy926 cells contained large cCCVs with few to moderate numbers of bacteria, predominantly LCVs and rarely SCVs (Figure 4(a)). Importantly, in contrast to the situation in normoxic *C. burnetii* infected murine BMDM, we did not detect aCCVs within normoxic EA.hy926 cells. These data indicate that normoxic EA.hy926 cells provide a more *C. burnetii-*friendly micromilieu. In hypoxic EA.hy926 cells, *C. burnetii* were detected only in aCCVs throughout the cells, as described for BMDM (Figure 4(b)). A membrane limiting aCCV was not always clearly detected (Figure 4(b)). The bacteria within aCCV had highly variable morphology, but the size was comparable to SCVs (Figure 4(f)). In detail, *C. burnetii* in normoxic cells had a mean diameter of 376 nm ±34 nm, while the diameter of bacteria in hypoxic cells was 210 nm ±21 nm (Figure 4(c)). In addition, a difference in the size and morphology of the periplasmic space was obvious between cells under normoxic and hypoxic conditions (Figure 4(d)). The majority of bacteria in normoxic endothelial cells were LCVs and, thus, had the characteristic electron lucent and irregularly dilated periplasmic space. Irrespective of their heterogeneous morphology, the periplasmic spaces of *C. burnetii* in hypoxic cells were comparable to those in SCVs (Figure 4(d,f)). The data indicate that the changes induced by hypoxia are more clearly seen in EA.hy926 cells (Figure 4) compared to BMDM (Figure 3). Importantly, further proliferation of *C. burnetii* was also halted by shifting the infected cells from a normoxic to a hypoxic environment (Figure S5).

Together, the data demonstrate that intracellular *C. burnetii* enter an SCV-like phenotype under hypoxic conditions, independently of the host species or cell type.

**Figure 5.**
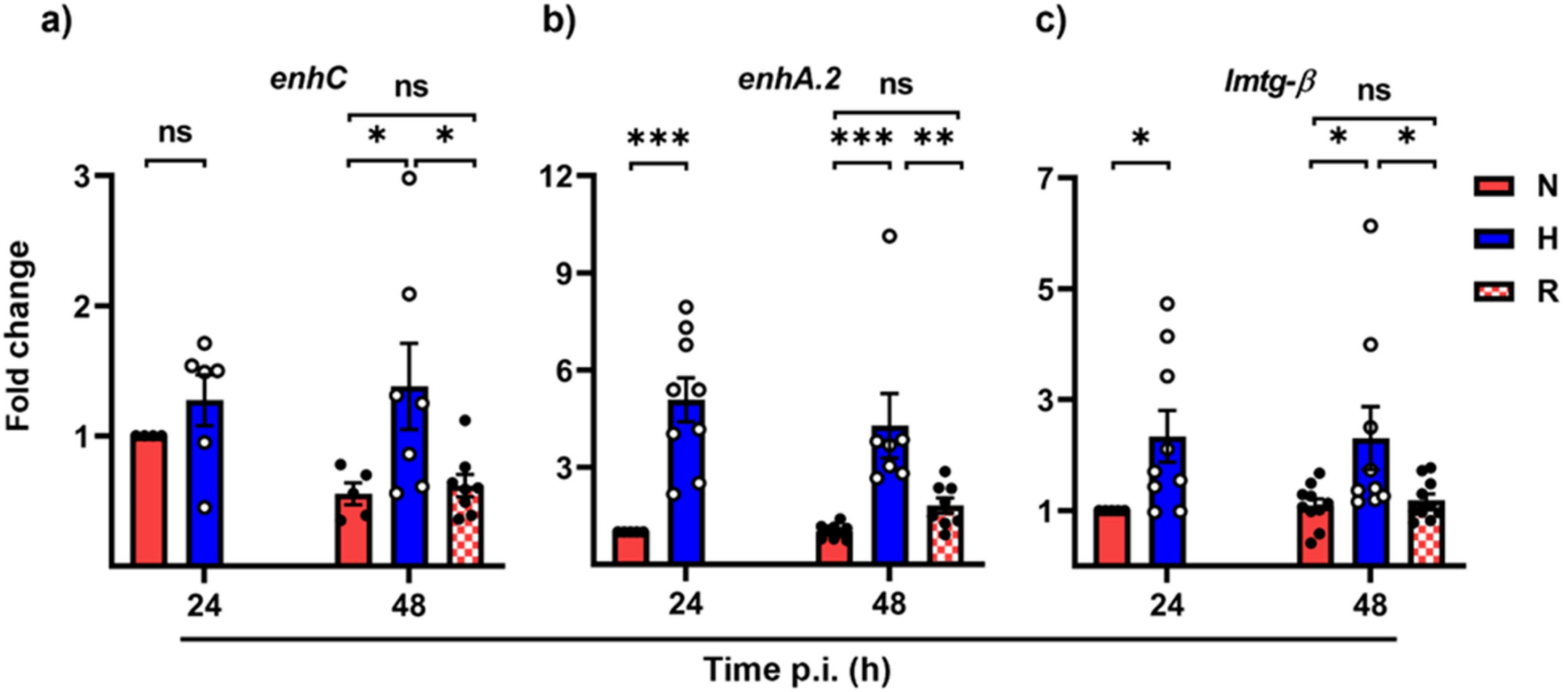
Hypoxia-mediated changes in infected BMDM induce upregulation of *C. burnetii* genes encoding cell wall remodeling proteins. Murine BMDM were infected for 48 h with *C. burnetii* at MOI 10 under normoxia (N) or hypoxia (H). Using qRT-PCR, the expression of the genes *enhC, enhA.2* and *lmtg-ß* involved in cell wall remodeling was determined. The data are depicted as mean ± SEM of 2^−ΔΔCT^ fold change values (using IS1111 as a calibrator) and normalized to the 24 h normoxic sample. Individual data points from 3–5 independent experiments performed in duplicates or triplicates are shown. One-sample t-test and Mann‑Whitney U test, ***p < 0.001, **p < 0.01, *p < 0.05, ns = not significant.

**Morphological changes of SCV-like persistent** ***C.*** ***burnetii*** **were accompanied by upregulation of cell wall remodeling enzymes.** The expression of genes encoding proteins involved in cell wall remodeling was shown to be upregulated during the late stationary phase, at a time point matching the transition to the SCV form [43], and in the SCV form [44]. Hence, the expression of such genes was analyzed to determine the underlying trigger of the morphological changes in the cell wall of SCV-like persistent *C. burnetii* in hypoxic BMDM. The expression of the YkuD domain protein containing gene *enhA.2* (CBU_0318), the lytic murein transglycosylase B encoding gene *lmtg-ß* (CBU_0925) and *enhC* (CBU_1136) were upregulated under hypoxic conditions (Figure 5). While *lmtg-ß* and *enhC* were only slightly, but significantly upregulated, *enhA.2* was upregulated ~5-fold under hypoxic conditions. Reoxygenation reset the hypoxia-induced upregulation of *enhA.2*, *lmtg-ß* and *enhC*, demonstrating the transient nature of this transcriptional regulation (Figure 5).

**Figure 6.**
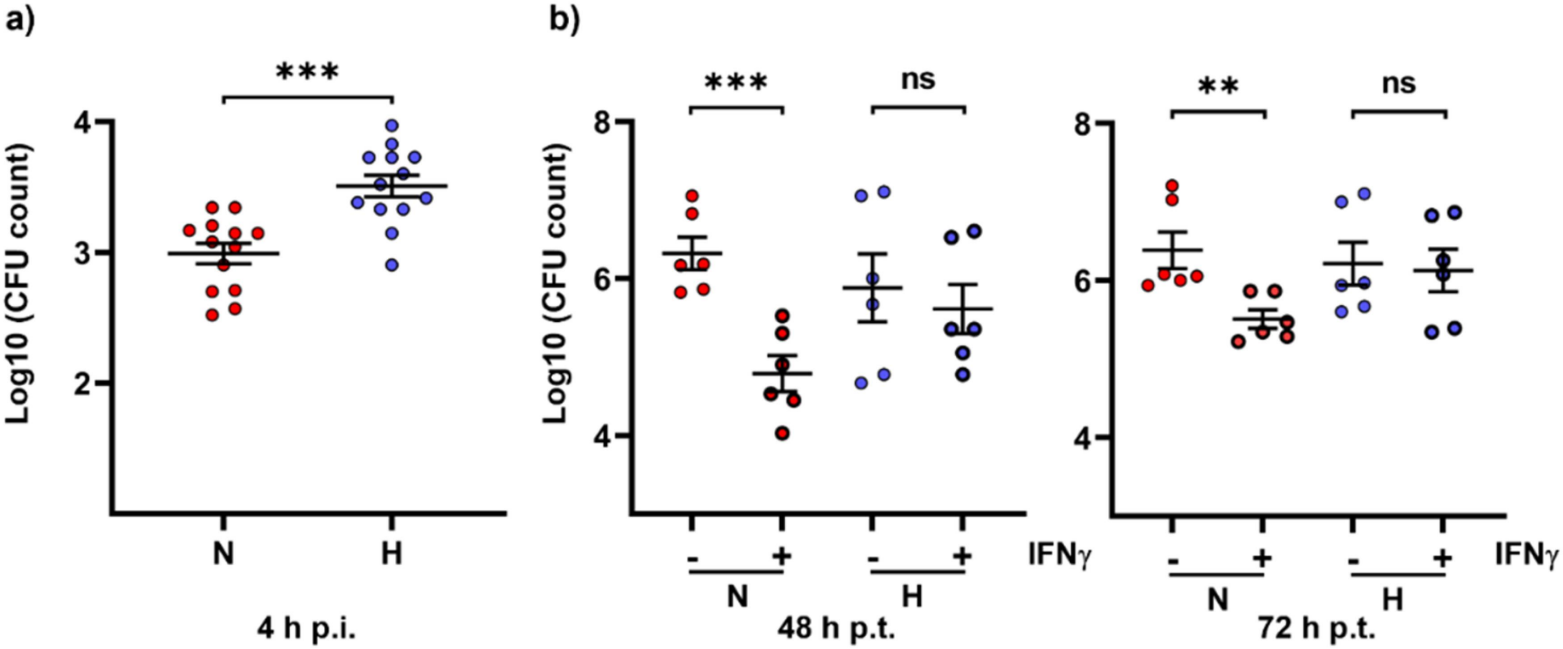
SCV-like persistent *C. burnetii* are characterized by increased infectivity. Murine BMDM were infected for 48 hours with *C. burnetii* at MOI 10 under normoxia (N) or hypoxia (H). a) *C. burnetii* were isolated and a CFU assay was performed to determine the amounts of viable bacteria. CHO cells were infected at MOI 3 for 4 h. The infection rate was determined by CFU counts. Data are presented as log10 of CFU counts. 3–4 independent experiments were performed in duplicates or triplicates. The data are depicted as means ± SEM. T-test, ***p < 0.0001. b) BMDM infected with *C. burnetii* for 48 h under normoxic (N) or hypoxic (H) conditions were treated with 10 ng/ml IFNγ for 48 and 72 h under normoxic conditions. A CFU assay was performed and survival was determined. Results from three independent experiments performed in duplicates are shown. Mann-Whitney U and t-test, ***p < 0.001, **p < 0.01, ns = not significant.

**The SCV-like persistent** ***C.*** ***burnetii*** **are more infectious and resistant to IFNγ treatment.** The SCV is often reported as the infectious form of *C. burnetii* [43]. Consequently, the infectivity of bacteria isolated from normoxic or hypoxic BMDM was determined. CHO cells were infected for 4 hours with *C. burnetii* isolated from normoxic or hypoxic BMDM. The infection rates were determined by CFU assay (Fig. S6). The number of intracellular bacteria was higher when CHO cells were infected with *C. burnetii* isolated from hypoxic BMDM (Figure 6(a)). This result indicates that SCV-like persistent *C. burnetii* are more infectious.

Cell autonomous control is important to control and clear the infection. It was shown that IFNγ-activation results in efficient cell autonomous control of a *C. burnetii* infection [45,46]. Therefore, the consequence of IFNγ activation on the survival of *C. burnetii* in normoxic and hypoxic BMDM was analyzed. Importantly, the infected normoxic and hypoxic BMDM were transferred to normoxic conditions for IFNγ treatment. While treatment with IFNγ resulted in killing of intracellular *C. burnetii* by previously normoxic BMDM over time, this was not observed in BMDM kept previously under hypoxic conditions (Figure 6(b)). These data suggest that the SCV-like persistent bacteria are less sensitive to IFNγ-activated cell autonomous control.

**Figure 7.**
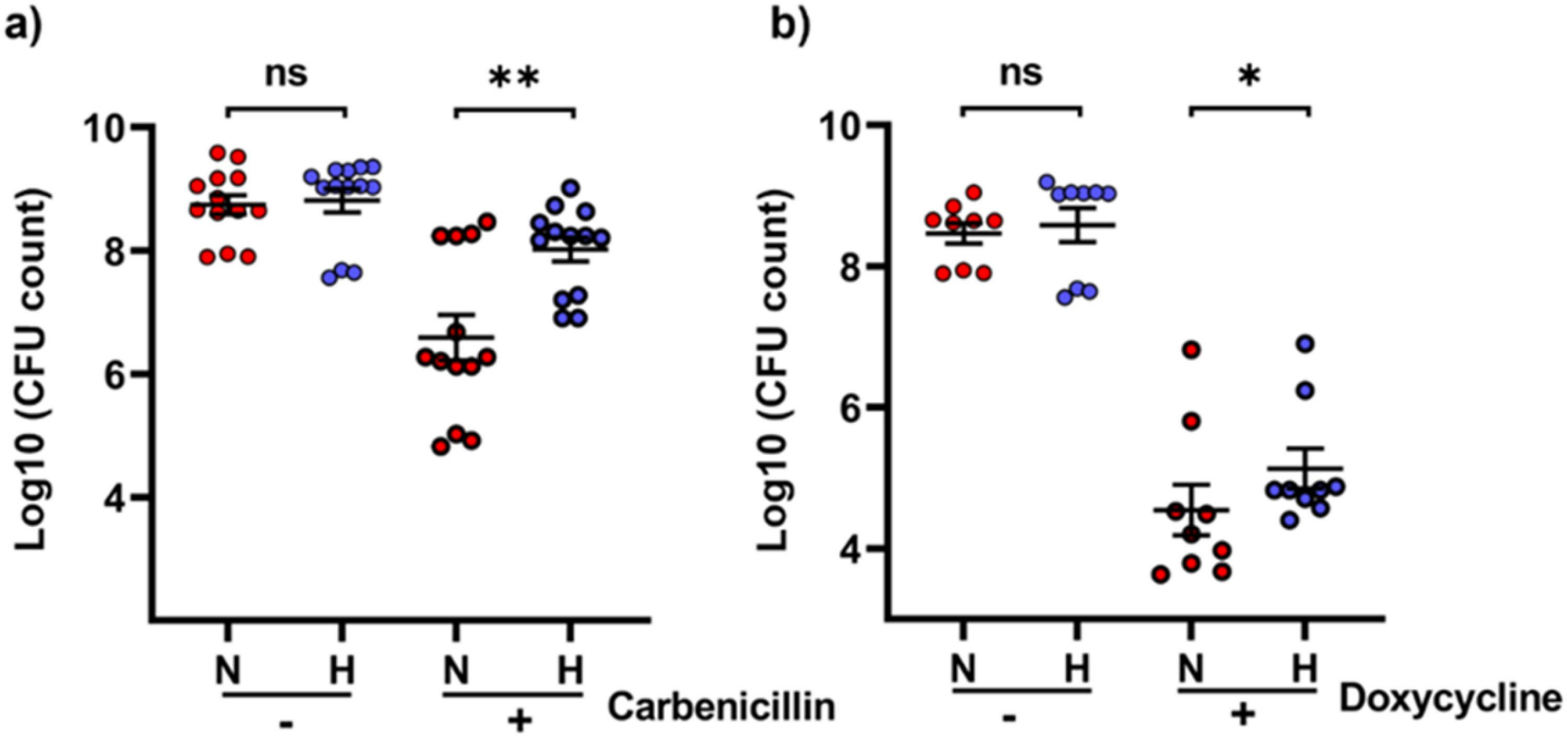
SCV-like persistent *C. burnetii* are more antibiotic-tolerant. Murine BMDM were infected with *C. burnetii* at MOI 10 under normoxia (N) or hypoxia (H). At 48 h post-infection *C. burnetii* were isolated and treated with a) 150 µg/ml carbenicillin or b) 3 ng/ml doxycycline in ACCM-2 medium for 7 days at 2.5% O_2_, 5% CO_2_ and 37°C. CFU was analysed from 4 independent experiments for carbenicillin and 3 independent experiments for doxycycline treatment, each experiment was performed in duplicates. Data are depicted as log10 values (mean ± SEM). Mann‑Whitney U test, **p < 0.01, *p < 0.05, ns = not significant.

**The SCV-like persistent** ***C.*** ***burnetii*** **are characterized by increased tolerance to carbenicillin and doxycycline.** Persistent bacteria become refractory to antibiotic treatment [47]. Therefore, the antibiotic susceptibility of isolated *C. burnetii* was investigated. Treatment of bacteria isolated from normoxic and hypoxic BMDM was chosen to prevent the influence of the host cell on the susceptibility of *C. burnetii*. Isolated *C. burnetii* were treated in axenic culture with either doxycycline or carbenicillin [48]. Doxycycline was chosen as it is the first-line treatment of acute Q fever. Carbenicillin was used as it has been shown that a thinner cell wall/outer membrane complex resulted in increased susceptibility to this antibiotic [26], suggesting that SCV-like persistent *C. burnetii* might be more tolerant to carboxypenicillin. Indeed, *C. burnetii*, isolated from hypoxic BMDM, are more resistant to treatment with carbenicillin (Figure 7(a)), but also slightly, but significantly, to doxycycline (Figure 7(b)). These data indicate that the morphological changes observed in the SCV-like persistent *C. burnetii* resulted in reduced susceptibility to the antibiotics doxycycline and carbenicillin.

## Discussion

Bacterial persistence is a cause of recurring and relapsing infections, as persistent bacteria are not sufficiently cleared by the host immune system [10]. This can be due to a lack of recognition by the immune system, mediated by, e.g., cell wall modulation [49]. In addition, persistent bacteria are often insensitive toward antibiotics, which prevents successful treatment [50]. This allows the bacteria to survive and subsequently expand once the cause of persistence is resolved, leading to concomitant disease progression.

Our knowledge about *C. burnetii* persistence is limited, although the long time-span between infection and disease outbreak indicates that *C. burnetii* persistence might be an important step toward chronic Q fever. A few reports have suggested that *C. burnetii* may persist in bone marrow [21] or in adipose tissue [22]. Importantly, at both sites the oxygen level might be reduced [51,52]. This and the fact that *C. burnetii* survives, but does not replicate in hypoxic macrophages [29], prompted us to investigate the physiological state of these bacteria in more detail. Of note, the low level of oxygen (0.5% O_2_) did not prevent axenic replication of *C. burnetii* (Figure 1(a)). Thus, the fact that *C. burnetii* was unable to replicate in hypoxic BMDM did not depend on oxygen limitation *per se*, but rather on hypoxia-induced modulation of host cell signaling and metabolism. In fact, the addition of citrate to *C. burnetii* infected hypoxic BMDM resulted in bacterial replication [29].

The oxygen level in the tissue differs from organ to organ and might already be low under resting conditions, e.g. in the bone marrow [53]. Infections with microbes might lead to increased reduction of available oxygen, which might be caused by the pathogen, but also by the host [54–56]. The limited availability of oxygen influences the metabolism of immune cells, which, consequently, also influences the host-pathogen interaction [57]. Thus, macrophages switch from oxidative phosphorylation to anaerobic glycolysis [58], the unfolded protein response (UPR) is activated and autophagy is induced [59]. Macrophage immune functions, such as inflammasome activation, phagocytosis, and antigen presentation, are also modulated [60–62]. These changes can either be beneficial or detrimental for the pathogen [57].

Nutrient limitation triggers the stringent response in bacteria to reprogram from rapid growth to metabolic homeostasis [10]. Although we observed a lack of the TCA cycle metabolite citrate as being responsible for the growth defect of *C. burnetii* in hypoxic BMDM [29], we did not observe transcriptional upregulation of the stringent response under hypoxic conditions (Figure 2(a)). However, while citrate was shown to prevent sporulation of *Bacillus subtilis* [63], it is unclear whether citrate limitation by itself triggers the stringent response in Gram-negative bacteria. In addition, the stringent response is only partially controlled at the transcriptional level. Hence, further research will be required to precisely define the role of citrate and the stringent response in *C. burnetii* persistence.

However, we did observe a general stress response (Figure 2(b)) and increased expression of *scvA* (Figure 2(c)) in *C. burnetii* within hypoxic BMDM.

Specifically, the upregulation of *clpA* and *degP.1*, which are involved in the general stress response, might have important implications for the host-pathogen interaction. Thus, the *Legionella pneumophila* type IV coupling protein DotL is destabilized and degraded in a ClpA-dependent manner [64], indicating that ClpA might influence the activity of the T4BSS. In addition, ClpA activity might be important for adaptation to and survival at higher temperatures [65,66], and for virulence [67]. DegP.1 was shown to be involved in the adaptation to environmental stresses and in virulence in *Edwardsiella piscicida* [68], and to be critical for acid resistance in *Escherichia coli* [69]. Further research is required to determine the role of ClpA and DegP.1 in *C. burnetii* persistence.

While the function of ScvA in *C. burnetii* is not completely known, it is an important marker for the development of SCVs. The expression of *scvA* seems to be at least partially dependent on RpoS function, as less *scvA* was transcribed in an RpoS mutant [26]. However, under hypoxic conditions *rpoS* is downregulated, while *scvA* is upregulated (Figures. 2(a,c)), suggesting that other factors also regulate its expression. Noteworthy, *C. burnetii* enters an SCV-like form under hypoxic conditions (Figure 2(c), 3 , and 4). So far, the SCV has been described as the extracellular stable form required for environmental transmission [2,39]. The intracellular transition to the SCV occurs in the stationary phase, approximately six days post-infection [70], a time point correlating with bacterial egress [71]. Our data suggest that the SCV is not only important for the extracellular phase of the pathogen, but also to survive adverse intracellular conditions. Interestingly, the characteristics of the CCVs containing the SCV-like persistent *C. burnetii* differ quite significantly from those of the classical CCV. The aCCVs were more pleomorphic and smaller in size. The fact that multiple aCCVs were present in one cell (Figure 3(b) and 4(b)) indicates that aCCVs did not undergo homotypic fusion. While the underlying reason is unknown, it can be speculated that the SCV-like persistent bacteria did not assemble the T4BSS [72], which translocates effector proteins involved in homotypic fusion events [73,74]. Indeed, the T4BSS was shown to be absent in SCVs, but present in LCVs using cryo-electron tomography analysis [72]. Using this technique, it was shown that SCVs are characterized by equidistant stacks of the inner membrane [28,72]. Earlier studies using TEM also identified internal membranous intrusions in SCVs [2]. We did not observe these structures. The underlying reason is unclear. In our study, routine TEM preparation of ultrathin sections modified by adding ruthenium red, a polycationic dye, to the fixatives to get better contrast of polysaccharides in the bacterial wall was performed. The chemical fixation and dehydration result in a variable degree of tissue shrinkage, while cryo-electron tomography allows imaging without dehydration of the samples. Thus, interpretation of observations in routine TEM preparations can be refined by the information from cryo EM investigations. This may explain the different results, but it might also be possible that SCVs and SCV-like persistent bacteria are morphologically and functionally different.

The precise regulation underlying the morphological changes observed (Figure 3 and 4) remains unknown. It has been speculated that ScvA plays a role in chromatin condensation, which protects DNA from stress-induced insults [39]. Consequently, the DNA condensation observed in the SCV-like persistent *C. burnetii* (Figure 3(f) and 4(f)) might be due to upregulation of *scvA* (Figure 2(c)). However, whether the transcriptional upregulation of *scvA* correlates with increased ScvA protein levels and, thus, protein function has to be clarified. Similarly, the alterations in the cell wall morphology observed in SCV-like persistent *C. burnetii* (Figure 3(d,f) and 4(d,f)) might be caused by increased expression of *enhC*, *enhA.2* and/or *lmtg-ß* (Figure 5). Further research will be required to disentangle the signaling cascade(s) leading to modulation of bacterial morphology.

These changes might cause rigidity of the cell wall, allowing *C. burnetii* to survive either host cell autonomous defense (Figure 6(b)) or antibiotic treatment (Figure 7(a,b)). Specifically, the modulation of the cell wall structure might be relevant for the latter. Peptidoglycan, the major component of the bacterial cell wall, is a net-like heteropolymer made of glycan chains crosslinked by short peptides [75,76]. Transpeptidases, including penicillin-binding proteins (PBPs), are enzymes that crosslink the peptidoglycan chains. The majority of peptide crosslinks are of the 4,3 type and are catalyzed by DD-transpeptidases [76]. However, bacteria also encode for LD-transpeptidases, which present a YkuD-like domain, and produce a 3,3 crosslink [77]. Both crosslink types serve to strengthen the cell wall. In *Escherichia coli*, the LD-transpeptidases are upregulated in the stationary phase and under stress conditions [78]. EnhA.2 is a YkuD-like domain-containing protein [43] and is upregulated under intracellular hypoxic conditions (Figure 5). Importantly, the SCV contains a higher percentage of 3,3 crosslinks [43,44], indicating that upregulation of cell wall remodeling enzymes (Figure 5), including EnhA.2, might result in increased 3,3 crosslinking. Bacteria containing 3,3 crosslinks were shown to be more resistant to beta-lactams [79] and to have improved cell envelope integrity [78]. Thus, the increased 3,3 crosslinks might result in improved stability of the cell wall, which in turn might provide improved antibiotic tolerance (Figures. 7(a,b)). Further investigations are necessary to prove this assumption. This might be relevant as the switch from 4,3 to 3,3 crosslinking might be a new therapeutic target to improve antibiotic susceptibility of this obligate intracellular pathogen. As the five *C. burnetii* LD transpeptidases (*cbu0053*, *cbu0318*, *cbu0957*, *cbu1138* and *cbu1394*) were specifically upregulated in SCVs [44], these genes might represent potential targets.

## Materials and methods

### Reagents

Chemicals were purchased from Sigma Aldrich unless indicated otherwise.

### Mice

C57BL/6 wild-type male mice (week 6–8) were obtained from Charles River Laboratories.

### Isolation and differentiation of murine bone marrow derived macrophages

Femurs and tibiae were isolated from C57BL/6 wild-type mice. The bone marrow cavity was flushed with cMoAb medium containing RPMI 1640 (Thermo Fisher), 10% fetal calf serum (FCS), 1% HEPES (AppliChem), 0.5% ß-mercaptoethanol using a 10 ml syringe fitted with a 27 G needle. The cells were centrifuged at 365 g for 10 min. The pellet was resuspended in macrophage differentiation medium (MDM) composed of DMEM-GlutaMAX (Gibco), 15% FCS, 20% supernatant from L929 cells, 1% penicillin/streptomycin (P/S, Gibco), 1% non‑essential amino acids (NEAA, Gibco) and 0.5% HEPES. 7 × 10^6^ cells were seeded per T75 culture flasks (Thermo Fisher) and differentiated for 7 days at 37°C and 5% CO_2_. The culture was supplemented with an additional 10 ml MDM on day 4.

### Cells and cell culture

To harvest the differentiated BMDMs, the culture medium was removed and ice-cold PBS added. Cell scrapers were used to detach the cells from the culture bottle. The cell suspension was centrifuged at 365 g for 10 min and the pellet resuspended in cMoAb medium at a concentration of 5 × 10^5^ cells/ml.

The human endothelial cell line EA.hy926 (ATCC®CRL-2922^™^) was cultivated in DMEM-GlutaMAX and 5% FCS. The CHO-FcR cells were cultivated in DMEM/F-12 (Thermo Fisher) and 5% FCS.

### Culture of *Coxiella burnetii*

*Coxiella burnetii* NMII clone 4 was inoculated at a concentration of 10^6^/ml in ACCM-2 (Sunrise Science Products) and cultivated for five days at 37°C, 5% CO_2_ and 2.5% O_2_. The bacterial culture was centrifuged at 4309 g for 20 min, and the pellet resuspended in PBS. The bacterial concentration was determined spectrophotometrically at OD_600_, with an OD of 1 corresponding to 10^9^ bacteria per ml. For infection experiments, a multiplicity of infection (MOI) of 10 was used for BMDMs, while an MOI of 200 was used for EA.hy926 cells.

### Hypoxia

For maintaining a hypoxic environment, experiments were performed within an InvivO_2_ hypoxic chamber (Baker Ruskinn) and the Whitley H35 Hypoxystation (Don Whitley Scientific Limited, UK) to control oxygen levels. Cells were treated under a defined hypoxic environment of 0.5% O_2_, 5% CO_2_, and 37°C. To ensure complete deoxygenation, all buffers and media were pre-incubated in the hypoxic chamber for a minimum of 4 h before being applied to the hypoxic cells.

### Infection of BMDM

The BMDM were infected with *C. burnetii* at MOI of 10 for 4 h under normoxic (21% O_2_, 5% CO_2_ and 37°C) and hypoxic (0.5% O_2_, 5% CO_2_ and 37°C) conditions. Afterward, the cells were washed thrice with 1x PBS to eliminate extracellular bacteria. The cells were then cultured for an additional 48 h in fresh pre-incubated cMoAb medium.

### Infection of EA.hy926 cells

5 × 10^4^ EA.hy926 cells/ml were seeded one day prior to infection with *C. burnetii* at MOI of 200. The infected cells were incubated for 6 h under normoxic (21% O_2_, 5% CO_2_ and 37°C) or hypoxic conditions (0.5% O_2_, 5% CO_2_ and 37°C). Afterward, the cells were washed six-times with 1x PBS to eliminate extracellular bacteria. The EA.hy926 cells were subsequently cultured for an additional 42 h in fresh, pre-incubated DMEM-GlutaMAX medium supplemented with 5% FCS.

### RNA extraction, cDNA synthesis, and qRT-PCR

The cells were lysed using 500 µl TRIzol reagent. RNA isolation was performed using the Direct-zol™ RNA Mini Prep Plus kit (Zymo research) according to the manufacturer’s instructions. To remove contamination of genomic DNA, a DNase treatment was performed using the RapidOut DNA removal kit (Thermo Fisher Scientific), according to the manufacturer’s instructions. cDNA was synthesized from 500 ng to 1 µg purified RNA by using the High-Capacity cDNA Reverse Transcription Kit (Applied Biosystems), according to the manufacturer’s instructions. Quantitative real-time PCR (qRT-PCR) was carried out using the PowerUp™ SYBER™ Green Master Mix (Applied Biosystems), following the manufacturer’s instructions. The cDNA was diluted 1:5 (~20 ng) and the primers (100 nM final concentration) listed in Table 1 were used. The expression levels of the genes were analyzed and referenced to the insertion element IS1111 and normalized to the 24 h normoxic sample. The 2^−ΔΔCT^ method was used to calculate the fold change.

**Table 1.** Primers used.

Name	CBU number	Sequence 5´- 3´
IS1111___fwd2		AATTTCATCGTTCCCGGCAG
IS1111_rev2		GCCGCGTTTACTAATCCCCA
ScvA_fwd	CBU_1267.1	TGGAAAGACAAAATGTCCAACAAC
ScvA_rev		GACGTGGGTTAGAAGCACC
SpoT_fwd2	CBU_0303	ATGCGGTTCACACCGATGTAGG
SpoT_rev2		GGATTCGGACGACCACTGGAAG
RelA_fwd13	CBU_1375	GGAAGAATGGTTAGCTACTAC
RelA_rev185		TCGCTATCGCAGTGCAAGG
ClpP_fwd105	CBU_0738	GCAGGTGGAAGACCACATG
ClpP_rev288		CTGCCCGATACACAATGTTC
ClpX_fwd574	CBU_0739	CCCTCGTTAACACGCGATG
ClpX_rev751		GAAGATCCGCGAAAGCGCC
ClpA_fwd250	CBU_1196	CAGCGAGTATTGCAGCGGG
ClpA_rev434		CGCGGCTTTGTTATGCCTTG
DksA_fwd3	CBU_1696	GGCGGTATCGAAAGCGATG
DksA_rev162		CCGTAGAGTCGACCTCTTC
RpoS_fwd2	CBU_1669	CTGGATGAACGTCATCGTGAGGTC
RpoS_rev2		GCGTAATTGCTGCAGAGCGTC
EnhC_fwd2	CBU_1136	TCACCGACTGGATTGCACCG
EnhC_rev2		GAGAAACCGTCTCTCTTGCGGG
EnhA.2_fwd1	CBU_0318	CCTATGCGTTAACGCTGGGTGAC
EnhA.2_rev1		GGGCATCAGCGATTAACCCATAGG
LMTG-ß_fwd2	CBU_0925	CCCTCCAGTTGGCATAATTACGCTG
LMTG-ß_rev2		TGATGGGCTTTCCAACCGTGTTTC
DegP.1_fwd2	CBU_0176	CCAGCATCAAAGAAGCGCTCAG
DegP.1_rev2		GGTTATCCACCTGCGAACCGTA
CsrA-2_fwd1	CBU_1050	CCAGACGTATCGGTGAATCGGTAATC
CsrA-2_rev1		GCCAATTTCTCCTGTTGAATCCGC

### Colony forming unit assay (CFU)

The medium was removed and the infected cells washed thrice with 1x PBS. The cells were shocked with 1 ml of ice-cold ddH_2_O and kept at 4°C for 30 min. Cell lysis was executed by vigorous pipetting. The bacteria were pelleted by centrifugation at 20,817 g for 1 min and the pellet resuspended in 200 µl PBS. A 10-fold serial dilution was carried out until a dilution factor of 10^−5^ was reached. From each dilution, 10 µl were spotted in triplicates onto ACCM-2/agarose plates containing 0.3% agarose. The plates were incubated for 8–10 days at 2.5% O_2_, 5% CO_2_ at 37°C. Colonies were counted microscopically.

### IFNγ treatment

BMDM were infected with *C. burnetii* for 48 h under normoxic and hypoxic conditions. The medium was replaced with fresh medium with or without 10 ng/ml IFNγ. The cells were incubated under normoxic conditions for 48 h and 72 h. A CFU assay was performed to determine the number of viable bacteria.

### Infectivity assay

Bacteria were isolated 48 h post-infection from normoxic and hypoxic BMDM by osmotic lysis. In detail, the infected cells were washed thrice with PBS, shocked with 1 ml of ice-cold ddH_2_O for 10 min at RT and 30 min at 4°C. Cell lysis was executed by vigorous pipetting. The bacteria were pelleted by centrifugation at 20,817 g for 1 min and the pellet resuspended in 200 µl PBS. A CFU assay was performed to determine the amount of viable *C. burnetii*. Isolated bacteria from each condition were used to infect CHO cells at MOI 3 for 4 h. A CFU assay was performed to evaluate the infection rate.

### Antibiotic treatment

Isolated bacteria were resuspended in 300 µl of 1x ACCM-2. 100 µl each was added to 4 ml 1x ACCM-2 medium with and without antibiotic (3 ng/ml doxycycline or 150 µg/ml carbenicillin). After 7 days of incubation at 2.5% O_2_, 5% CO_2_ at 37°C, a CFU assay was performed.

### Electron microscopy sample preparation (EM)

#### *Coxiella burnetii* in ACCM-2

*Coxiella burnetii* in ACCM-2 were fixed with an equal volume of 2.5 % glutaraldehyde in 0.1 M cacodylate buffer (pH 7.2) containing 1.8 % glucose for 8 h at 4°C. Then, the fixative was replaced by cacodylate buffer.

For negative contrast preparation, the suspension was vortexed and a 30 µl-droplet placed on a dental wax plate. 300-mesh copper grids filmed with formvar, coated with carbon, and hydrophilized by glow discharge were incubated on the droplet for 30 min. After washing with distilled water, they were floated on droplets of 0.5 % and 0.2 % uranyl acetate for 1 min for contrasting. Grids were examined in a transmission electron microscope (Tecnai 12, FEI Deutschland GmbH, Dreieich, Germany) and representative micrographs of 5 grid squares were taken with a digital camera at 2900x magnification (TEMCAM FX416, TVIPS, Gauting, Germany). The diameter of 100 bacteria was measured using the EM-Measure software (TVIPS).

For resin embedding, the remaining suspension was centrifuged at 21,000 g for 30 min. The supernatant was discarded and the pellet mixed with 2% molten agarose on a glass slide, allowed to cool and sectioned into 1 mm^3^ cubes. Cubes were post-fixed in 2% osmium tetroxide and embedded in Araldite Cy212. Relevant areas were selected in Toluidine-blue-stained semi-thin sections. Ultrathin sections (80 nm) collected on 200 mesh copper grids were stained with uranyl acetate and lead citrate and examined by transmission electron microscopy (Tecnai 12; FEI/ThermoFisher Scientific) at 80 kV. Representative electron micrographs were taken with a digital camera (TEMCAM FX416, TVIPS, Garching, Germany).

#### BMDM and EA.hy926 cells

For electron microscopy, 1 × 10^6^ BMDMs and 2 × 10^5^ EA.hy926 cells were seeded in 6-well plates. The cells were infected at MOI of 10 (BMDMs) and 200 (EA.hy926) for 48 h under both hypoxic and normoxic conditions. 48 h post infection, the cells were fixed by adding 5 ml of ice-cold ruthenium red-glutaraldehyde fixative in 0.1 M cacodylate buffer (pH 7.3, 2.5% glutaraldehyde (Roth, Karlsruhe), 2% paraformaldehyde (Roth, Karlsruhe), 0.075% ruthenium red (Serva, Heidelberg), cacodylate acid (Roth, Karlsruhe)) per well and incubated for 3 h at 4°C. Gentle agitation was applied intermittently during fixation to ensure uniform exposure. The fixative was removed, and the cells washed three times for 15 min with ice-cold 0.1 M cacodylate buffer. The cells were carefully scraped from the wells and centrifuged at 1500 g for 5 min at 4°C. The cell pellets were resuspended and stored in cacodylate buffer at 4°C until further processing.

Samples were centrifuged at 1500 g for 5 min, the supernatant discarded and the resulting cell pellets mixed with a drop of liquid 2% agarose (50 °C) (Roth, Karlsruhe). After cooling, the agarose containing the cells was cut into 2 mm^3^ blocks and transferred to cacodylate buffer (pH 7.3). The agarose blocks were embedded in araldite CY212 (Plano, Wetzlar) using a tissue processor (Lynx II, Electron Microscopic Sciences, Hatfield, USA). A 2 h treatment with osmic acid-ruthenium red solution (2% osmic acid in aqua bidest (Roth, Karlsruhe), 0.06% ruthenium red in 0.1 M cacodylate buffer) was included before dehydration for post-fixation of lipid membranes. Acetone (Roth, Karlsruhe) was used for dehydration. The incubation steps were performed under vacuum and with mild agitation over 28 h. At the end, the agarose blocks were transferred to tissue molds with freshly prepared araldite CY212 and cured at 50°C for 48 h and at 65°C for 24 h. Excessive resin was trimmed off (Leica-EM-Trim, Leica, Wetzlar). Semi-thin sections of 0.3 µm thickness were cut on a ultramicrotome (Leica Ultracut UCT, Leica, Wetzlar) using a diamond knife (Histo, Diatome, Nidau, Switzerland) and stained with 0.25% toluidine blue. They were used to select defined areas for ultrathin sectioning. Ultra-thin sections of 80 nm thickness were cut on an ultramicrotome using a diamond knife (Ultra 35°, Diatome, Nidau, Switzerland), collected on 300-mesh copper grids (Plano, Wetzlar) and contrasted with lead citrate (Leica, Wetzlar) and uranyl acetate (Plano, Wetzlar) using an automatic stainer (Leica EM AC20, Leica, Wetzlar). Samples were examined in a transmission electron microscope (TECNAI 12, FEI Deutschland, Dreieich) at an acceleration voltage of 80 KV, and findings documented using a digital camera (TEMCAM FX416, TVIPS, Gauting). Measurements were performed on the ultra-micrographs using the EM-Measure software (version from 2021–05-19, TVIPS, Gauting). Micrographs were optimized for brightness and contrast using Adobe Lightroom® (Adobe CS, Version 2025). Image sections were selected, scale bars set using Adobe Indesign® (Adobe CS, Version 2025). Final images were served as sRGB-tif files using Photoshop® (Adobe CS, Version 2025).

### Statistical analysis

Statistical analyses were conducted using GraphPad Prism (version 10). Statistical details can be found in the legends of the figures. Normality distribution was tested with D’Agostino & Pearson normality test or the Kolmogorov-Smirnov test, or, when *N* < 8, the Shapiro-Wilk normality test or the Kolmogorov-Smirnov test. For non-normally distributed datasets, the Mann-Whitney U test was used. Otherwise, the unpaired two-tailed Student’s t-test was used. A one-sample t-test was used when comparing datasets to normalized values. A value of *p* < 0.05 was considered significant.

## Supplementary Material

Asghar et al_Supplemenary information.docx

## Data Availability

The data that support the findings of this study are openly available in figshare at https://doi.org/10.6084/m9.figshare.30674672 [80].

## ARRIVE checklist

This study adheres to the ARRIVE guidelines.
