# Supplementary material for: Coxiella burnetii establishes a small cell variant (SCV)-like persistent form to survive adverse intracellular conditions
Source: Virulence. 2026 Sep 10;17(1):2707792. doi: 10.1080/21505594.2026.2707792 (PMC13568565; doi:10.1080/21505594.2026.2707792)
Supplement: Supplementary file 1 [file KVIR_A_2707792_SM7497.docx]

**Supplementary information**

**Figure S1**

**Figure S1. *C. burnetii* infect equally well regardless whether they were axenically grown at 2.5% or 0.5% O_2_.** *C. burnetii* were grown axenically at 2.5% or 0.5% O_2_ for 5 days. EA.hy926 cells were infected with these *C. burnetii* at MOI 200 for 6 h. Bacteria were isolated from infected cells and a CFU assay was performed. Three technical experiments were performed and the data are shown as mean ± SEM.

**Figure S2**

**Figure S2. At 48 h post infection normoxic (N) and hypoxic (H) BMDMs harbour equal amounts of *C. burnetii*.** Murine BMDMs were infected for 48 h with *C. burnetii* at MOI 10 under normoxia (N) or hypoxia (H). *C. burnetii* were isolated and a CFU assay was performed. The CFU/ml was calculated. Four independent experiments were performed in triplicates. The data are shown as individual data points (mean ± SD). Significance was determined by a Mann-Whitney U test; ns=not significant.

**Figure S3**

**Figure S3. Hypoxic BMDM contain lipid droplets and peroxisomes**. Murine BMDMs were infected for 48 h with *C. burnetii* at MOI 10 under hypoxia. The cells were fixed with ruthenium red-glutaraldehyde, embedded in araldite, and ultrathin sectioned for electron microscopy. An ultrathin section of a hypoxic BMDM is shown. Lipid droplets are marked with **L,** and peroxisomes with **P**. The scale bar = 1 µm.

**Figure S4**

**Figure S4. BMDMs contain mainly aCCVs.** Murine BMDM were infected for 48 h with *C. burnetii* at MOI 10 under normoxia. 350-400 CCVs in total were classified as either classic (cCCV) or atypical (aCCV) from three independent experiments. Classical CCVs represent approximately 5% and atypical CCVs roughly 95%. Significance was determined by a paired t-test; ***p<0.001.

**Figure S5**

**Figure S5. The further proliferation of *C. burnetii* stops under hypoxic conditions.** The EA.hy926 cells were infected with *C. burnetii* at MOI 200 for 24, 48 and 96 h under normoxia (N) or for 48 h under normoxia (N) followed by 48 h under hypoxia (H). A correlation to *C. burnetii* numbers were determined using qRT-PCR of the IS1111 gene. Two independent experiments were performed in triplicate. The data are shown as mean CT value ± SD. Mann-Whitney U test, **p<0.01, *p<0.05.

**Figure S6**

**Figure S6.** **Experimental set up of the reinfection experiment**. Murine BMDMs were infected for 48 h with *C. burnetii* at MOI 10 under normoxia (N) or hypoxia (H). *C. burnetii* were isolated and a CFU assay was performed. CHO cells were infected with *C. burnetii* at MOI 3 for 4 h. To determine the amount of intracellular *C. burnetii* a CFU assay was performed. This figure was created in BioRender (VI29LR8K0N).
